# Detection of *Sarcocystis* parasites in environmental samples from Lithuanian farms^☆^

**DOI:** 10.1016/j.fawpar.2025.e00267

**Published:** 2025-05-08

**Authors:** Agnė Baranauskaitė, Petras Prakas, Modestas Petrauskas, Selene Rubiola, Elena Servienė, Živilė Strazdaitė-Žielienė

**Affiliations:** a State Scientific Research Institute Nature Research Centre, Akademijos st. 2, Vilnius 08412, Lithuania; b Department of Veterinary Sciences, University of Turin, Grugliasco, Torino 10095, Italy

**Keywords:** *Sarcocystis*, Environmental samples, Zoonotic transmission, Molecular detection, *cox1*

## Abstract

Most studies on apicomplexan *Sarcocystis* spp. in domestic animals have primarily focused on examining animal carcasses using both morphological and molecular methods. However, to accurately assess the risk of *Sarcocystis* infections in livestock and to develop effective prevention strategies, it is essential to investigate the environmental reservoirs of these parasites. The aim of this study was to identify *Sarcocystis* species with domestic animals as intermediate hosts by analysing environmental samples (water, hay, and soil) collected from Lithuanian farms and to compare their occurrence across different sample types. In total, 90 environmental samples were collected over 3 years and analysed for the presence of *Sarcocystis* spp. using nested polymerase chain reactions targeting the *cox1* gene. The results indicated that livestock are most likely to acquire infections via the ingestion of contaminated water or feed, while soil posed a lower risk of transmission. An assessment of species distribution across sampled farms revealed that the type of livestock raised did not influence the diversity of *Sarcocystis* spp. Notably, at least six of seven target species (*S. arieticanis*, *S. bertrami*, *S. bovifelis*, *S. capracanis*, *S. cruzi*, *S. miescheriana*, *S. tenella*) were detected at least once on eight of 10 farms. Additionally, two zoonotic *Sarcocystis* species, *S. hominis* and *S. suihominis*, were identified in environmental samples. This study emphasises the potential risk of livestock infection through contaminated environmental and feed sources and highlights the critical role of environmental monitoring in preventing the transmission of *Sarcocystis* spp. to farm animals.

## 1 Introduction

Members of the genus *Sarcocystis* are single-celled protozoans that can infect reptiles, birds, and mammals, including humans. They have an obligatory two-host prey–predator life cycle. Sarcocysts are formed mainly in the muscle tissues of intermediate hosts, whereas oocysts sporulate in the small intestines of the definitive host. Intermediate hosts become infected with these parasites through food or water contaminated with sporocysts, while definitive hosts (predators or scavengers) are infected by ingesting animal tissues containing mature sarcocysts (Dubey et al., 2016).

The most thoroughly studied species of *Sarcocystis* include those with sarcocysts found in the muscle tissues of domestic animals. The extent of infection varies depending on geographical location, animal housing and feeding, and sanitary conditions. Although natural outbreaks are rare, large numbers of sporocysts can lead to various health problems. *Sarcocystis* spp. transmitted by dogs (such as *S. arieticanis*, *S. capracanis*, *S. cruzi*, and *S. tenella*) can cause severe symptoms of sarcocystosis in domestic animals (Dubey et al., 2016; Sykes et al., 2011).

Humans can become infected with intestinal sarcocystosis through the consumption of raw or undercooked beef (*S. hominis*, *S. heydorni*, and *S. sigmoideus*) or pork/wild boar meat (*S. suihominis*) (Hu et al., 2016; Pacifico et al., 2023; Moniot et al., 2025). Muscular sarcocystosis in humans is acquired through food or water contaminated with faeces containing sporocysts of *S. nesbitti* or potentially other *Sarcocystis* spp. (Fayer et al., 2015; Lau et al., 2014). Acute muscular sarcocystosis in humans may cause myositis, fever, itchy rashes, painful muscle swelling, and weakness (Dubey, 2015). Meanwhile, infection with intestinal sarcocystosis causes nausea, loss of appetite, vomiting, stomach pain, diarrhoea, and even tachycardia. Most cases of *Sarcocystis* infection have been recorded in tropical and subtropical countries of Asia and Southeast Asia, as well as in various parts of Europe, and such cases are often associated with close and frequent contact between humans and animals in unsanitary conditions (Fayer, 2004; Fayer et al., 2015). However, because of misidentification of disease symptoms and the lack of appropriate detection methods, sarcocystosis is being neglected on a global scale. To date, epidemiological data on sarcocystosis are still scarce because studies on zoonotic *Sarcocystis* spp. are mainly conducted by analysing cattle or pig/wild boar carcasses (Chauhan et al., 2020; Huang et al., 2019; Pacifico et al., 2023; Prakas et al., 2020), while faecal samples from children or adults are less frequently tested for this disease (Moniot et al., 2025; Nimri, 2014; Rubiola et al., 2020; Van Den Broucke et al., 2023), and zoonotic species have been rarely tested in environmental samples (Shahari et al., 2016). The following species of *Sarcocystis* infecting domestic animals have been detected and studied most frequently in Europe: *S. arieticanis* and *S. tenella*, forming sarcocysts in muscles of sheep; *S. bertrami*, producing sarcocysts in horses; *S. bovifelis*, *S. cruzi*, *S. hirsuta*, and *S. hominis*, which are considered to be specific for cattle; *S. capracanis*, which is the most common species found in goats; and finally *S. miescheriana* and *S. suihominis*, which are two known species for pigs, although the latter is still rarely found (Gjerde et al., 2020; Marandykina-Prakienė et al., 2023; Šneideris et al., 2024).

To date, most research on *Sarcocystis* spp. has been conducted by analysing animal carcasses using morphological or molecular methods (Castro-Forero et al., 2022). However, such post-mortem studies do not allow for the prediction of possible routes of infection or the prevention of transmission to animals and humans. By contrast, only a few environmental studies worldwide have identified *Sarcocystis* parasites (Baranauskaitė et al., 2023; Shahari et al., 2016; Strazdaitė-Žielienė et al., 2022). *Sarcocystis* species found in environmental samples cannot be distinguished using morphological methods because the sizes of sporocysts from different *Sarcocystis* species overlap. Therefore, the objective of our work was to use molecular methods to identify zoonotic *Sarcocystis* species and those with domestic animals as intermediate hosts in environmental water, hay, and soil samples, and to compare the observed prevalence of these parasites.

## 2 Materials and methods

### 2.1 Sample collection

Over three years (2022–2024), environmental water (*n* = 30), hay (n = 30), and soil (n = 30) samples were collected during the warm season, in July and August, when animals are kept outside in the fields. Moreover, there is relatively little rainfall during the summer period, which could otherwise affect data from hay and soil samples. With farmers' consent, 10 livestock farms from across Lithuania were selected for the study; these farms raised cows, bulls, sheep, goats, or horses (**Table S1**). Considering the varying moisture and structural properties of hay and soil on each farm, volumetric measurements were used for all sample types. On each study farm, 1 L of hay, 200 mL of soil, and 1 L of water were collected near the livestock grazing area. Each type of sample was collected from three different locations on the farm and then combined into one to increase the probability of detection. Samples were collected in sterile 1 L glass flasks using sterile gloves or a metal scoop, depending on the sample type. Sample collection was coordinated with the farmers, and the collected samples were transported in portable coolers with ice packs. Samples were stored at 4 °C before further processing and were analysed within 1–2 days of collection.

### 2.2 Preparation of environmental samples and extraction of genomic DNA

A methodology optimised in a previous study (Strazdaitė-Žielienė et al., 2022), based on the principle of filtration through different pore size filters (1 mm pore metal sieve, Whatman™ Qualitative Filter Paper Grade 4, and MF-Millipore® 5 μm pore membrane), was used to collect and concentrate sporocysts of *Sarcocystis* spp. in the examined samples. The filter paper and membrane were pre-sterilised and used individually for each sample, while the metal sieve was rinsed with water after each filtration, then sterilised with 70 % ethanol and finally autoclaved. Before filtration, hay and soil samples were poured with 1 L of sterile distilled water and washed by shaking for 1 h at 110 rpm at room temperature. After washing, the liquid was decanted and used for filtration. The concentrated 2 mL sample was stored at 4 °C and subsequently used for genomic DNA extraction using the GeneJET Genomic DNA Purification Kit (Thermo Fisher Scientific Baltics, Vilnius, Lithuania), according to the manufacturer's recommendations.

### 2.3 Molecular identification of *Sarcocystis* species

During this study, samples were analysed for the presence of nine selected *Sarcocystis* species (*S. arieticanis*, *S. bertrami*, *S. bovifelis*, *S. capracanis*, *S. cruzi*, *S. hominis*, *S. miescheriana*, *S. tenella*, and *S. suihominis*) that form sarcocysts in meat-producing animals. For the detection of DNA from these species, nested polymerase chain reactions (PCRs) were performed using species-specific primers targeting the cytochrome *c* oxidase subunit I (*cox1*) gene. The composition of the PCR mixtures and reaction conditions followed those described in a previous study (Baranauskaitė et al., 2023), with the exception of the detection of two zoonotic species, *S. hominis* and *S. suihominis*, for which primers were developed in this study. To detect DNA of *S. hominis*, the primer pairs GaHoEF (5′-TCTCTGGTTTTGGTAACTACTTCGT-3′) / GaHoER (5′-CAGACACTGGGATATAATACCGAAC-3′) and GaHoEF2 (5′-CATTGGCTGGACTCTCTATGCT-3′) / GaHoER2 (5′-AAATATCGGCAGGGTAATTATCAA-3′) were used for the first and second steps of PCR, respectively. Meanwhile, for the detection of *S. suihominis*, primers V2su5 (5′-CAACGTGTACTTTACCATGCAC-3′) / V2su6 (5′-AGCCGGGCAGAATCAGAATA-3′) and V2su7 (5′-GTATGGCTAATCCACTCCGTAA-3′) / V2su8 (5′-GCATCATAAAAACCAAAGTTGAG-3′) were used. Positive controls (DNA of tested *Sarcocystis* species) and negative controls (nuclease-free water instead of template DNA) were included to ensure the reliability of results. PCR amplicons were visualised by electrophoresis on a 1 % agarose gel. *Sarcocystis arieticanis*, *S. bertrami*, *S. bovifelis*, *S. capracanis*, *S. cruzi*, *S. miescheriana*, and *S. tenella* were detected using a previously established methodology (Baranauskaitė et al., 2023), and to confirm these results, only three PCR fragments of each species were sequenced. By contrast, all PCR products amplified with *S. hominis* and *S. suihominis* species-specific primers were sequenced. Sequencing was performed using the Big-Dye® Terminator v3.1 Cycle Sequencing Kit (Thermo Fisher Scientific Baltics) and the 3500 Genetic Analyzer (Applied Biosystems, Foster City, CA, USA). The obtained *cox1* sequences of *Sarcocystis* species were deposited in GenBank under the accession numbers PV033819–PV033840.

### 2.4 Data analysis

Statistical analysis and species diversity estimations were performed using Python (v 3.12.4). A chi-square test was conducted with SciPy (Virtanen et al., 2020) to examine parasite detection by sample type. Shannon diversity indices were calculated using SciPy's entropy function, with data management via pandas (McKinney, 2010) and numerical operations using NumPy (Harris et al., 2020). To evaluate intraspecific and interspecific genetic differences, the *cox1* sequences generated in the present study were analysed using the nBLAST sequence similarity search algorithm (Altschul et al., 1990). The selection of a nucleotide substitution model and phylogenetic analysis using the maximum likelihood method was carried out with MEGA v.11.0.13 (Tamura et al., 2021). Multiple sequence alignments were generated using the MUSCLE algorithm incorporated into MEGA. The bootstrap method with 1,000 replicates was used to test the robustness of the phylogeny.

## 3 Results

### 3.1 Comparison of *Sarcocystis* environmental DNA (eDNA) detection across various environmental samples

Comparison of the obtained *cox1* sequences amplified with species-specific primers confirmed the detection of eDNA of *S. arieticanis*, *S. bertrami*, *S. bovifelis*, *S. capracanis*, *S. cruzi*, *S. miescheriana*, and *S. tenella* in the analysed environmental samples. In each case, the estimated intraspecific and interspecific genetic differences did not overlap (**Table S2**). The results showed statistically significant differences (χ^2^ = 16.92, df = 2, *p* = 0.00021) in the prevalence of *Sarcocystis* spp. eDNA among sample types, with the highest prevalence observed in water (31.9 %), followed closely by hay (31.0 %), and the lowest in soil (16.7 %) (Table 1). A summary analysis estimated that on average, eDNA of five different *Sarcocystis* spp. was most frequently found in water and hay samples, while three were found in soil samples. Analysis of individual farms (F) revealed some differences in species diversity across the examined ecosystems. For example, in F1, species diversity in the water sample was three times higher than in the hay, and only one species was found in the soil. Furthermore, no *Sarcocystis* eDNA was detected in the soil of the second and third farms, although three to seven species were identified in water and hay samples. Data on the animals kept on each examined farm, feeding and housing conditions, and the prevalence of *Sarcocystis* spp. are provided in **Table S1**. The results indicate that the most frequently identified species on an individual farm is not necessarily the one whose intermediate hosts are raised there.

**Table 1:** The detection of *Sarcocystis* spp. in water, hay, and soil samples during a three-year period. The overall frequency was determined by summing the total number of PCR-positive samples and dividing by the maximum possible number of positive cases, which was calculated as 3 study years × 10 farms × 7 species, resulting in 210 potential positive cases for each study environment.

	Number of positive cases										Overall frequency	
Farm	F1	F2	F3	F4	F5	F6	F7	F8	F9	F10	Total	Total, %
Water	7	5	9	7	4	10	7	11	4	3	67	31.9
Hay	2	4	7	5	7	7	6	10	8	9	65	31.0
Soil	1	0	0	7	8	3	5	5	3	3	35	16.7

After assessing the prevalence of *Sarcocystis* spp. on individual farms, regardless of the material examined, it was found that most often, three (33.3 %) different *Sarcocystis* spp. were identified on a single farm within the same survey year. Somewhat less frequently, five (16.7 %) or even six (13.3 %) different species were found on the same farm. In rare cases, no *Sarcocystis* eDNA was detected (6.7 %), or only one (10.0 %), four (10.0 %), or two (6.7 %) species were identified. Moreover, when comparing species abundance on the same farm across different survey years, notable fluctuations in prevalence were observed. For example, only one species was detected on farms F5, F9, and F10 in 2024, 2023, and 2024, respectively, whereas in other years, as many as six or seven different species were identified (Table 2). However, even in years with low species diversity, the cumulative data over the 3-year period showed that six to seven different *Sarcocystis* spp. were detected on most of the farms studied (8 of 10).

**Table 2:** Number of different *Sarcocystis* species identified in individual farms in different study years. The total detection of *Sarcocystis* spp. indicates the overall number of different species detected over a three-year period, since the same species may have been detected for several years, the total is not a three-year cumulative figure. The Shannon Diversity Index (H) is given in the brackets.

	F1	F2	F3	F4	F5	F6	F7	F8	F9	F10
2022	5 (2.25)	3 (1.52)	6 (2.52)	5 (2.25)	7 (2.64)	3 (1.52)	6 (2.52)	5 (2.25)	5 (2.25)	6 (2.52)
2023	0 (0.00)	3 (1.52)	3 (1.52)	4 (1.91)	6 (2.52)	3 (1.52)	3 (1.52)	5 (2.25)	1 (0.00)	3 (1.52)
2024	3 (1.52)	0 (0.00)	2 (1.00)	2 (1.00)	1 (0.00)	4 (1.91)	3 (1.52)	3 (1.52)	4 (1.91)	1 (0.00)
Total	6 (2.45)	4 (1.83)	6 (2.60)	5 (2.08)	7 (2.65)	6 (2.43)	7 (2.68)	7 (2.42)	6 (2.37)	6 (2.21)

### 3.2 Distribution of *Sarcocystis* spp. in water, hay, and soil samples

**Fig. 1:**
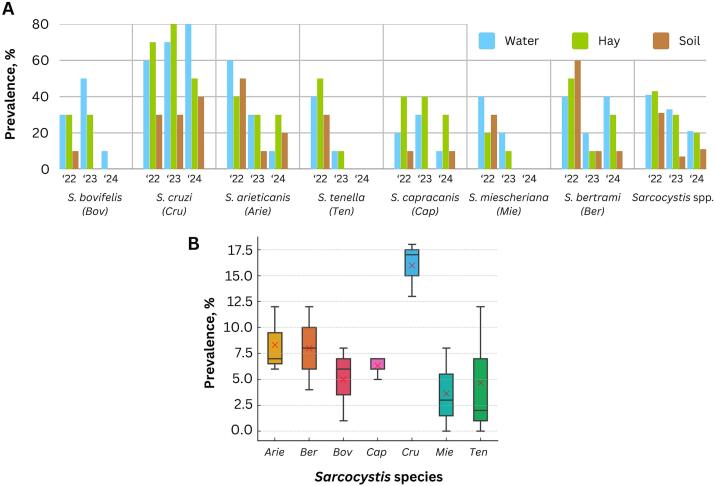
Prevalence of *Sarcocystis* spp. in environmental samples. **A.** Distribution of species in water, hay, and soil samples during the study period. The overall frequency of *Sarcocystis* spp. was calculated by summing up all positive samples and dividing them by the total number of samples tested. **B.** Boxplot analysis showing detection count distributions of *Sarcocystis* species. Boxes represent the interquartile range (25th–75th percentiles), horizontal lines within boxes indicate the median, and red “×” markers denote the mean detection counts. Whiskers extend to the minimum and maximum values within 1.5 times the interquartile range. (For interpretation of the references to colour in this figure legend, the reader is referred to the web version of this article.)

During the study, most *Sarcocystis* species were generally found in water samples, with similar or slightly lower frequencies in hay, and least frequently in soil. The occurrence of *S. cruzi* and *S. capracanis* exhibited minimal variation across sample types. The cattle-infecting *S. bovifelis* was primarily detected in the first and second years of the study, with only one positive water sample recorded in the third year. The sheep-infecting *S. arieticanis* was mostly detected in the first year and appeared half as frequently in the second and third years, while *S. tenella* was found in only two samples during the second year and was not detected at all in the third. Furthermore, the detection of *S. miescheriana*, which infects pigs, declined threefold each year. Although the occurrence of *S. bertrami*, infecting horses, was three times lower in the second year than in the first, the number of detections doubled in the third year (Fig. 1A). Analysis of detection counts obtained from eDNA revealed distinct profiles across *Sarcocystis* species. *Sarcocystis cruzi* was the most prevalent, with detection counts ranging from 13 to 18 per year and a mean of 16.0, exceeding those of all other species and highlighting its consistent presence over time. Intermediate frequencies were observed for *S. arieticanis* and *S. bertrami*, with means of 8.33 and 8.0, respectively, followed by *S. capracanis* with a mean of 6.33. By contrast, *S. bovifelis*, *S. miescheriana*, and *S. tenella* displayed lower detection frequencies, with means of 5.0, 3.6, and 4.6, respectively (Fig. 1B).

### 3.3 Detection of zoonotic *Sarcocystis* spp. in environmental samples

To date, the development of primers for the detection of zoonotic *Sarcocystis* species has been hindered by the limited availability of accurate reference data in GenBank. However, during the second year of this study, primers specifically tailored for the detection of the zoonotic species *S. hominis* in environmental water, hay, and soil samples were successfully developed. This zoonotic *Sarcocystis* species was subsequently detected in both the second and third years of the study, in 4 and 7 of 10 farms, respectively. In 2023, *S. hominis* was detected three times in water, four times in hay, and once in soil samples, resulting in an overall prevalence of 26.7 %. In the third year of the study, the overall prevalence increased slightly to 33.3 %, with detections occurring four times in both water and hay samples and twice in soil samples. Notably, this study represents the first known detection of the zoonotic *S. hominis* in environmental samples worldwide.

**Fig. 2:**
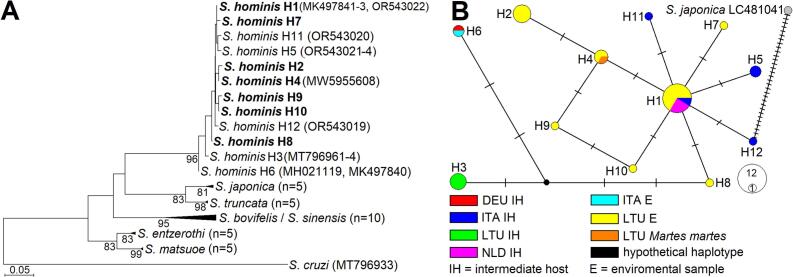
Phylogenetic relationships of *S. hominis*. **A.** The maximum likelihood phylogenetic tree based on 192 bp *cox1* sequences. The tree was rooted on *S. cruzi* and scaled according to the branch length. The Kimura 2 parameter + G evolutionary model was set for analysis. GenBank accession numbers are given in brackets. Overall, 12 different haplotypes (H1−H12) of *S. hominis* were distinguished. Bootstrap values higher than 70 are presented next to branches. Sequences of *S. hominis* generated in the present work are in boldface. **B.** The median joining haplotype network of *S. hominis*. The area of the circle represents the frequency of the haplotype. Mutational steps are indicated by dashes. DEU – Germany, ITA – Italy, LTU – Lithuania, NLD – the Netherlands.

Using PCR primers specific for *S. hominis*, we obtained eighteen 192 bp *cox1* sequences (**Table S3**). Based on BLAST analysis, these sequences showed 96.9 %–100 % similarity to *S. hominis* sequences in the database, and up to 83.8 %–84.0 % similarity to those of *S. bovifelis*, *S. entzerothi*, *S. japonica*, and *S. truncata*. In the maximum likelihood phylogenetic tree, the sequences obtained in the present study were placed with high support (96) alongside those of *S. hominis* and were genetically distinct from other *Sarcocystis* spp. (Fig. 2A). Based on the short 192 bp *cox1* fragment analysed, *S. hominis* was most closely related to *S. japonica* and *S. truncata*. Thus, both BLAST and phylogenetic analysis confirmed the presence of *S. hominis* in 18 environmental samples. In this study, we identified seven *cox1* haplotypes of *S. hominis*, differing by one to three single-nucleotide polymorphisms (Fig. 2B). In total, 12 *S. hominis* haplotypes have been determined across several European countries. The most common haplotype was detected in Italy, the Netherlands, and Lithuania; haplotypes in the network have not been grouped according to their geographical origin (Prakas et al., 2020; Rubiola et al., 2024; Rubiola et al., 2018).

**Fig. 3:**
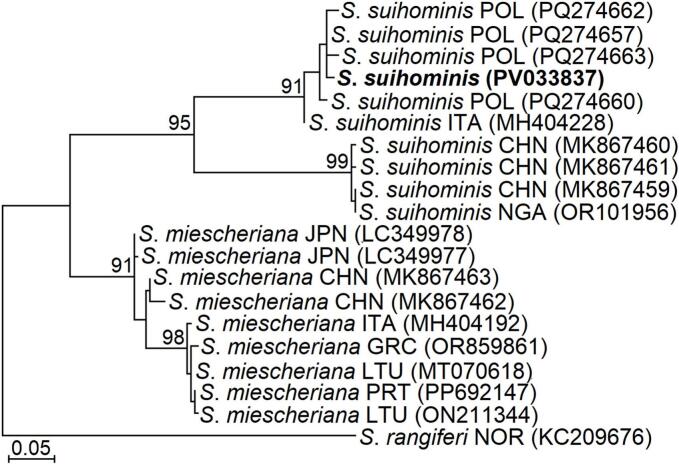
The phylogenetic placement of *S*. *suihominis* identified in the environmental sample from Lithuania. The tree was constructed using the maximum likelihood method, rooted on *S*. *rangiferi,* and scaled according to the branch length. GenBank accession numbers are shown in brackets. Numbers next to branches indicate bootstrap values higher than 70. The sequence of *S*. *suihominis* obtained in the present study is highlighted in bold. CHN – China, GRC – Greece, ITA – Italy, JPN – Japan, LTU – Lithuania, NGA – Nigeria, NOR – Norway, POL – Poland, PRT – Portugal.

During the second year of the study, primers were also developed for identification of the zoonotic *S. suihominis*. Using the V2su5 and V2su6 primers, we obtained a single *S. suihominis* sequence, which was isolated from environmental water samples collected on farm F5. The analysed 293-bp sequence showed 97.3 %–99.3 % similarity to *S. suihominis* sequences from Poland and Italy, 83.5 % similarity to those from China and Nigeria, and 81.2 %–83.6 % similarity (with 93 % query coverage) to *S. miescheriana*. In the phylogenetic tree, all *S. suihominis* sequences available in GenBank were divided into two evolutionarily distinct clusters (Fig. 3). The *S. suihominis* sequence from Lithuania grouped with those originating from Europe, while sequences from China and Nigeria formed a separate cluster.

## 4 Discussion

To date, comprehensive studies analysing environmental samples across various ecosystems have not been conducted, primarily because many protozoan infections are believed to be predominantly waterborne. Nevertheless, sporocysts excreted into the environment via faeces can spread throughout the area due to weather or mechanical factors (Pepper et al., 2014). Furthermore, in cases of sarcocystosis infection, common signs such as fever, nausea, and diarrhoea typically appear first, often leading to confusion with other bacterial or viral illnesses (Fayer et al., 2015). Generally, sarcocystosis continues to be overlooked because of insufficient awareness among farmers and food industry workers. Consequently, it is imperative to monitor and prevent the spread of parasitic species in natural environments, particularly those involving meat-producing animals as intermediate hosts. The present study aimed to identify selected zoonotic *Sarcocystis* spp. and species with domestic animals as intermediate hosts in environmental water, hay, and soil samples, and to compare their prevalence across different natural settings. The findings of the 3-year study indicated that the highest likelihood of animal infection occurs through water or hay, while the lowest contamination with sporocysts was observed in soil samples. However, such results may be influenced by the survival of sporocysts in different soil types. A long-term study conducted in Australia revealed that an increase of one pH unit in soil corresponded to a 14 % reduction in the density of *Sarcocystis*-positive farms (Taggart et al., 2019). Thus, it appears that animals are more likely to become infected with sporocysts through water or feed, not only because these are integral components of the animals' diet, but also due to natural factors affecting sporocyst survival in different environmental conditions.

Previous research has demonstrated that the concentration of *Sarcocystis* spp. in natural environments is relatively low, necessitating the use of sensitive methods for accurate detection of these parasites. The primary objective of this study was to optimise the methodology to enable its use without reliance on costly techniques or specialised knowledge. Nested PCR, using two pairs of species-specific primers, was selected to evaluate the occurrence and diversity of *Sarcocystis* species in Lithuanian livestock farms. Although nested PCR is not as rapid and straightforward as direct PCR, it is more accessible and cost-effective than quantitative PCR (Kim et al., 2011). Moreover, regardless of the species, the life cycle stage of these parasites present in the environment exhibits uniform size and morphological characteristics, precluding the possibility of conducting analytical evaluation procedures such as spiking. Consequently, to ensure the specificity and suitability of the methodology, the detection capabilities via nested PCR were assessed using various dilutions of DNA isolated from the corresponding species' sarcocyst. Notably, real-time PCR has been infrequently applied in the studies of *Sarcocystis* to date (Moré et al., 2013). The widespread application of this method is hindered by the lack of suitable primers, necessitating the development of new gene markers for precise species differentiation. Therefore, efforts are being made in this direction to apply quantitative PCR for the detection of a broad spectrum of *Sarcocystis* spp. in the near future. Consequently, nested PCR was used to detect eDNA of *Sarcocystis* parasites during this study. Species-specific primers were chosen for species previously detected during both morphological and molecular studies in Lithuania (Baranauskaitė et al., 2023; Marandykina-Prakienė et al., 2022; Prakas et al., 2020). Although cattle-infecting *S. hirsuta* was detected in 30.4 % of cattle carcass samples (Prakas et al., 2020), in a study of Lithuanian environmental water bodies it was detected in only 3 of 150 tested water bodies (Baranauskaitė et al., 2023). For these reasons, it was decided not to include *S. hirsuta* in this study. Based on nested PCR results, nine *Sarcocystis* spp. (*S. arieticanis*, *S. bertrami*, *S. bovifelis*, *S. capracanis*, *S. cruzi*, *S. hominis*, *S. miescheriana*, *S. tenella*, and *S. suihominis*) were identified over the 3 years (Fig. 1). The definitive hosts of the established *Sarcocystis* species include humans and predators of the Canidae and probably Mustelidae families (Šneideris et al., 2024). The prevalence of the detected species correlated with the results of previous studies of environmental water bodies conducted in Lithuania (Baranauskaitė et al., 2023; Strazdaitė-Žielienė et al., 2022). During this research, the most common parasites found in tested samples were *S. cruzi* (86.7 %), infecting cattle; *S. arieticanis* (60.0 %), infecting sheep; and *S. bertrami* (53.3 %), infecting horses. The detection of species across various individual farms demonstrates that the type of animals raised does not limit the diversity of *Sarcocystis* species. According to the results, on average (in 8 of 10 farms), at least six different species infecting domestic animals were identified during the study period. Additionally, six farms also had at least one zoonotic species identified in at least one type of environmental sample. It can be assumed that these parasites can enter livestock farms through various natural routes; for example, with dogs spending time and wandering outdoors, or with wild predators of the families Canidae and Mustelidae entering the farm territory. Moreover, the intermediate hosts of all detected species, except *S. miescheriana*, which is also carried by wild boars, are exclusively animals reared on private or commercial farms, as there are no free-ranging farm animals in Lithuania. Although the European bison (*Bison bonasus*) is recognised as a potential intermediate host for species infecting cattle, The Red Data Book of Lithuania (https://www.raudonojiknyga.lt/, accessed on 20 January 2025) indicates that the current population of bison is limited to 130 individuals living in central Lithuania, suggesting their minimal impact on the transmission of *Sarcocystis* spp. Moreover, the general overview of species detection showed that there is no obvious relationship between the frequency of *Sarcocystis* detection and livestock housing or feeding conditions.

Notably, this study marks the first global detection of the zoonotic *S. hominis*, which infects both cattle and humans, in environmental water, hay, and soil samples. The seven *cox1* haplotypes of *S. hominis* determined in this study significantly clustered with other haplotypes of this species previously detected in Germany, Italy, and the Netherlands (Fig. 2). DNA of this species was found in five of seven cattle-raising farms, highlighting a significant warning regarding the potential risk to both domestic animals and human health. Previous research on cattle carcasses in Lithuania reported a 13.7 % prevalence of *S. hominis* (Prakas et al., 2020), with a lower infection load than other species infecting cattle. Consequently, the detection of this species is complex, necessitating precise and sensitive methodologies. The selection of appropriate research conditions, genetic markers, and PCR primers is challenging because other species with a high infection load can interfere with both morphological and molecular studies. Therefore, the development of advanced molecular methods for species detection is essential to accurately assess the occurrence of this species in carcasses and in natural environments. To date, only one study has attempted to identify *S. hominis* using multiplex real-time PCR targeting the 18S rRNA gene (Moré et al., 2013). However, given that the 18S rRNA gene is deemed unsuitable for differentiating *Sarcocystis* species infecting cattle (Gjerde, 2016), and considering the limited data on the prevalence of this species at the time, *S. hominis* may have been misidentified in various previous studies. This study also identified another zoonotic species, *S. suihominis*, for the first time in environmental samples. Furthermore, *S. suihominis* was previously recorded in Lithuania over 30 years ago through microscopic examination (Prakas and Butkauskas, 2012). Although this species was found in only one water sample, it is important to note that *S. suihominis* is uncommon in this region (Calero-Bernal et al., 2016; Pacifico et al., 2023). Currently, combined morphological and molecular diagnosis of *S. suihominis* is lacking in Europe, as this species is typically identified solely through DNA analysis (Pacifico et al., 2023). By contrast, *S. suihominis* is predominantly found in Asian countries, where pigs are raised close to humans, often under unsanitary conditions (Banerjee et al., 1994; Chauhan et al., 2020; Huang et al., 2019; Saleque and Bhatia, 1991). In this study, the sequence of *S. suihominis* exhibited significant divergence (81.2 %–83.6 % similarity) from sequences obtained in China and Nigeria (Huang et al., 2019; Obadiah et al., 2024) (Fig. 3). Such variations within the *cox1* gene among *Sarcocystis* species from geographically distant regions are typically regarded as interspecific (Gjerde, 2013). Consequently, it is imperative to conduct studies that integrate molecular and morphological methods to more accurately evaluate the global infection rates and genetic diversity of *S. suihominis*. Moreover, morphological differentiation of *Sarcocystis* species based on their sporocyst/oocyst characteristics in definitive hosts or environmental samples is practically unfeasible (Dubey et al., 2016). Therefore, there is a pressing need to expand morphological investigations of zoonotic *Sarcocystis* species in intermediate hosts (Obadiah et al., 2024; Pacifico et al., 2023). In summary, the findings underscore the necessity for enhanced sanitary conditions in livestock farms. Although the detection of zoonotic species was not included in the initial year of the study, the obtained results indicate a substantial potential for both animals and humans to become infected with *Sarcocystis* parasites. Thus, further research is needed to conduct a more comprehensive search for *Sarcocystis* species and to evaluate the potential transmission routes of species atypical for the region.

## 5 Conclusions

This study represents the first comparative analysis of the prevalence and species richness of *Sarcocystis* spp. in three different types of environmental samples collected from farms. The highest overall detection frequency of *Sarcocystis* species was observed in water (31.9 %) and hay (31.0 %) samples, with the lowest in soil (16.7 %). Over the 3-year period, nine *Sarcocystis* spp. (*S. arieticanis*, *S. bertrami*, *S. bovifelis*, *S. capracanis*, *S. cruzi*, *S. hominis*, *S. miescheriana*, *S. tenella*, and *S. suihominis*), which infect both humans and animals, were identified from the collected samples. The most prevalent species were *S. cruzi* (56.7 %), *S. arieticanis* (31.1 %), and *S. bertrami* (30 %), which infect cattle, sheep, and horses, respectively. A moderate detection frequency was noted for the zoonotic *S. hominis*, identified in 18 of 60 samples (30.0 %). The prevalence of other species was below 30.0 %. The zoonotic *S. suihominis*, which circulates between pigs/wild boar and humans, was identified in only one water sample (1.7 %). Thus, the present study demonstrates that zoonotic *Sarcocystis* spp. and parasite species using livestock as intermediate hosts can be detected in farm environments, posing a risk to both animal and human health.

## CRediT authorship contribution statement

**Agnė Baranauskaitė:** Writing – original draft, Visualization, Software, Resources, Methodology, Investigation, Formal analysis, Data curation, Conceptualization. **Petras Prakas:** Writing – review & editing, Visualization, Software, Investigation, Data curation. **Modestas Petrauskas:** Investigation. **Selene Rubiola:** Writing – review & editing, Formal analysis. **Elena Servienė:** Writing – review & editing, Supervision, Resources, Project administration, Funding acquisition. **Živilė Strazdaitė-Žielienė:** Writing – review & editing, Supervision, Resources, Project administration, Methodology, Investigation, Funding acquisition, Conceptualization.

## Funding

The project “Comprehensive analysis of microorganisms and Protozoan parasites in farmlands: water, soil, and feed” has received funding from the 10.13039/501100004504Research Council of Lithuania (LMTLT), agreement (S-MIP-23–7).

## Declaration of competing interest

Agne Baranauskaite reports financial support was provided by Research Council of Lithuania. If there are other authors, they declare that they have no known competing financial interests or personal relationships that could have appeared to influence the work reported in this paper.

## Data Availability

Data supporting the conclusions of this article are included in the article. The sequences generated in the present study were submitted to the GenBank database under Accession Numbers PV033819–PV033840.

## Appendix A Supplementary data

Supplementary material Image 1

## References

[bb0005] Altschul S.F., Gish W., Miller W., Myers E.W., Lipman D.J. (1990). Basic local alignment search tool. J. Mol. Biol..

[bb0010] Banerjee P.S., Bhatia B.B., Pandit B.A. (1994). *Sarcocystis suihominis* infection in human beings in India. J. Vet. Parasitol..

[bb0015] Baranauskaitė A., Strazdaitė-Žielienė Ž., Servienė E., Butkauskas D., Prakas P. (2023). Molecular identification of protozoan *Sarcocystis* in different types of water bodies in Lithuania. Life.

[bb0020] Calero-Bernal, R., Pérez-Martín, J.E., Reina, D., Serrano, F.J., Frontera, E., et. al., 2016. Detection of zoonotic Protozoa *toxoplasma gondii* and *Sarcocystis suihominis* in wild boars from Spain. Zoonoses Public Health 63(5), 346–350. doi: 10.1111/zph.12243. 26604045

[bb0025] Castro-Forero S.P., Bulla-Castañeda D.M., López Buitrago H.A., Díaz Anaya A.M., Madeira De Carvalho L.M., Pulido-Medellín M.O. (2022). *Sarcocystis* spp., a parasite with zoonotic potential. Bulg. J. Vet. Med..

[bb0030] Chauhan R.P., Kumari A., Nehra A.K., Ram H., Garg R. (2020). Genetic characterization and phylogenetic analysis of *Sarcocystis suihominis* infecting domestic pigs (*Sus scrofa*) in India. Parasitol. Res..

[bb0035] Dubey J.P. (2015). Foodborne and waterborne zoonotic sarcocystosis. Food Waterborne Parasitol..

[bb0040] Dubey J.P., Calero-Bernal R., Rosenthal B.M., Speer C.A., Fayer R. (2016).

[bb0045] Fayer R. (2004). *Sarcocystis* spp. in human infections. Clin. Microbiol. Rev..

[bb0050] Fayer R., Esposito D.H., Dubey J.P. (2015). Human infections with *Sarcocystis* species. Clin. Microbiol. Rev..

[bb0055] Gjerde B. (2013). Phylogenetic relationships among *Sarcocystis* species in cervids, cattle and sheep inferred from the mitochondrial cytochrome c oxidase subunit I gene. Int. J. Parasitol..

[bb0060] Gjerde B. (2016). Molecular characterisation of *Sarcocystis bovifelis*, *Sarcocystis bovini* n. sp., *Sarcocystis hirsuta* and *Sarcocystis cruzi* from cattle (*Bos taurus*) and *Sarcocystis sinensis* from water buffaloes (*Bubalus bubalis*). Parasitol. Res..

[bb0065] Gjerde B., de la Fuente C., Alunda J.M., Luzón M. (2020). Molecular characterisation of five *Sarcocystis* species in domestic sheep (*Ovis aries*) from Spain. Parasitol. Res..

[bb0070] Harris C.R., Millman K.J., van der Walt S.J., Gommers R., Virtanen P. (2020). Array programming with NumPy. Nature.

[bb0075] Hu J.J., Wen T., Chen X.W., Liu T.T., Esch G.W., Huang S. (2016). Prevalance, morphology, and molecular characterization of *Sarcocystis heydorni* Sarcocysts from cattle (*Bos taurus*) in China. J. Parasitol..

[bb0080] Huang Z., Ye Y., Zhang H., Deng S., Tao J., Hu J., Yang Y. (2019). Morphological and molecular characterizations of *Sarcocystis miescheriana* and *Sarcocystis suihominis* in domestic pigs (*Sus scrofa*) in China. Parasitol. Res..

[bb0085] Kim D.M., Park G., Kim H.S., Lee J.Y., Neupane G.P. (2011). Comparison of conventional, nested, and real-time quantitative PCR for diagnosis of scrub typhus. J. Clin. Microbiol..

[bb0090] Lau Y.L., Chang P.Y., Tan C.T., Fong M.Y., Mahmud R., Wong K.T. (2014). Short report: *Sarcocystis nesbitti* infection in human skeletal muscle: possible transmission from snakes. Am. J. Trop. Med. Hyg..

[bb0095] Marandykina-Prakienė A., Butkauskas D., Gudiškis N., Juozaitytė-Ngugu E., Januškevičius V. (2022). Molecular identification of *Sarcocystis* species in sheep from Lithuania. Animals.

[bb0100] Marandykina-Prakienė A., Butkauskas D., Gudiškis N., Juozaitytė-Ngugu E., Bagdonaitė D.L. (2023). *Sarcocystis* species richness in sheep and goats from Lithuania. Vet. Sci..

[bb0105] McKinney W. (2010). Data structures for statistical computing in Python. Scipy.

[bb0110] Moniot M., Combes P., Costa D., Argy N., Durieux M.F. (2025). Simultaneous detection of *Sarcocystis hominis*, *S*. *Heydorni*, and *S*. *Sigmoideus* in human intestinal Sarcocystosis, France, 2021–2024. Emerg. Infect. Dis..

[bb0115] Moré G., Schares S., Maksimov A., Conraths F.J., Venturini M.C., Schares G. (2013). Development of a multiplex real time PCR to differentiate *Sarcocystis* spp. affecting cattle. Vet. Parasitol..

[bb0120] Nimri L. (2014). Unusual case presentation of intestinal *Sarcocystis hominis* infection in a healthy adult. JMM Case Reports.

[bb0125] Obadiah H.I., Wieser S.N., Nzelu I.N., Olaolu O.S., Jagab H.S. (2024). First molecular detection of *Sarcocystis suihominis* in a domestic pig of Nigeria. Parasitol. Res..

[bb0130] Pacifico L., Rubiola S., Buono F., Sgadari M., D’Alessio N. (2023). Molecular differentiation of *Sarcocystis miescheriana* and *Sarcocystis suihominis* using a new multiplex PCR targeting the mtDNA *cox1* gene in wild boars in southern Italy. Res. Vet. Sci..

[bb0135] Pepper I.L., Gerba C.P., Gentry T.J. (2014).

[bb0140] Prakas P., Butkauskas D. (2012). Protozoan parasites from genus *Sarcocystis* and their investigations in Lithuania. Ekologija.

[bb0145] Prakas P., Strazdaitė-Žielienė Ž., Januškevičius V., Chiesa F., Baranauskaitė A. (2020). Molecular identification of four *Sarcocystis* species in cattle from Lithuania, including *S*. *Hominis*, and development of a rapid molecular detection method. Parasites and Vectors.

[bb0150] Rubiola S., Chiesa F., Zanet S., Civera T. (2018). Molecular identification of *Sarcocystis* spp. in cattle: partial sequencing of cytochrome C oxidase subunit 1 (*COI*). Ital. J. Food Saf..

[bb0155] Rubiola S., Civera T., Ferroglio E., Zanet S., Zaccaria T. (2020). Molecular differentiation of cattle *Sarcocystis* spp. by multiplex PCR targeting 18S and COI genes following identification of *Sarcocystis hominis* in human stool samples. Food Waterborne Parasitol..

[bb0160] Rubiola S., Moré G., Civera T., Hemphill A., Frey C.F. (2024). Detection of *Sarcocystis hominis*, *Sarcocystis bovifelis*, *Sarcocystis cruzi*, *Sarcocystis hirsuta* and *Sarcocystis sigmoideus* sp. nov. in carcasses affected by bovine eosinophilic myositis. Food Waterborne Parasitol..

[bb0165] Saleque A., Bhatia B.B. (1991). Prevalence of *Sarcocystis* in domestic pigs in India. Vet. Parasitol..

[bb0170] Shahari S., Tengku-Idris T.I.N., Fong M.Y., Lau Y.L. (2016). Molecular evidence of *Sarcocystis nesbitti* in water samples of Tioman Island, Malaysia. Parasites and Vectors.

[bb0175] Šneideris D., Moskaliova D., Butkauskas D., Prakas P. (2024). The distribution of *Sarcocsytis* species described by ungulates-canids life cycle in intestines of small predators of the family Mustelidae. Acta Parasitol..

[bb0180] Strazdaitė-Žielienė Ž., Baranauskaitė A., Butkauskas D., Servienė E., Prakas P. (2022). Molecular identification of parasitic Protozoa *Sarcocystis* in water samples. Vet. Sci..

[bb0185] Sykes J.E., Dubey J.P., Lindsay L.L., Prato P., Lappin M.R. (2011). Severe myositis associated with *Sarcocystis* spp. infection in 2 dogs. J. Vet. Intern. Med..

[bb0190] Taggart P.L., Stevenson M., Firestone S.M., McAllister M.M., Caraguel C.G. (2019). Spatial analysis of a cat-borne disease reveals that soil pH and clay content are risk factors for sarcocystosis in sheep. Front. Vet. Sci..

[bb0195] Tamura K., Stecher G., Kumar S. (2021). MEGA11: molecular evolutionary genetics analysis version 11. Mol. Biol. Evol..

[bb0200] Van Den Broucke S., Dorny P., Van Esbroeck M., Bottieau E. (2023). Microscopic detection of intestinal Sarcocystis infection diagnosed in international travelers at the Institute of Tropical Medicine, Antwerp, Belgium, from 2001 to 2020. Am. J. Trop. Med. Hyg..

[bb0205] Virtanen P., Gommers R., Oliphant T.E., Haberland M., Reddy T. (2020). SciPy 1.0: fundamental algorithms for scientific computing in Python. Nat. Methods.

