# Supplementary material for: Detection of Sarcocystis parasites in environmental samples from Lithuanian farms
Source: Food Waterborne Parasitol. 2025 May 8;39:e00267. doi: 10.1016/j.fawpar.2025.e00267 (PMC12139677; doi:10.1016/j.fawpar.2025.e00267)
Supplement: Supplementary file 1 — Supplementary material [file mmc1.docx]

**Supplementary materials**

**Table S1.** Characteristics of farms selected for the study (detected *Sarcocystis* species theoretically using the same intermediate hosts that are kept on individual farms are indicated in bold).

| **Farm** | **Location** | **Animals kept in farm** | **Housing/feeding conditions** | **Detected species (number of samples detected)** |
| --- | --- | --- | --- | --- |
| **1** | Vilnius district | Horses | Kept in a **closed** area, next to a lake into which a river flows, but the horses can drink only during a walk with a rider; horses are fed both grass and stacked hay | *S*. *arieticanis* (water, hay, soil),  ***S*. *bertrami* (water)**,  *S*. *bovifelis* (2x water),  *S*. *cruzi* (water, hay),  *S*. *tenella* (water),  *S*. *miescheriana* (water) |
| **2** | Molėtai district | Bulls | Bulls are kept free in a large enclosure **with** **access** to a pond. They eat grass and stacked hay in the summer and are fed stacked hay during the winter | *S*. *bertrami* (water, hay),  *S*. *capracanis* (water, hay),  ***S*. *cruzi* (2x water, 2x hay)**,  *S*. *miescheriana* (water) |
| **3** | Mažeikiai district | Cows | Cows are kept free in a large meadow near a waterbody for extinguishing a fire, however **without access** to reach the water; cows eat grass in the summer from the meadow, and are fed stacked hay during the winter | *S*. *arieticanis* (water, hay),  *S*. *bertrami* (water, hay),  ***S*. *bovifelis* (2x water, hay)**,  *S*. *capracanis* (water),  ***S*. *cruzi* (3x water, 2x hay)**,  *S*. *miescheriana* (water, hay),  *S*. *tenella* (hay) |
| **4** | Telšiai district | Cows | Cows are kept free in a large meadow **with** **access** to the river; cows eat grass in the summer from meadow, and are fed stacked hay during the winter | *S*. *arieticanis* (2x water, hay, 2x soil),  *S*. *bertrami* (water, hay, 2x soil),  ***S*. *cruzi* (2x water, 2x hay, 3x soil)**,  *S*. *miescheriana* (2x water),  *S*. *tenella* (hay) |
| **5** | Zarasai district | Cows | Cows are kept free in a large area **with** **access** to the river that flows into a pond; cows eat grass in the summer, and are fed stacked hay during the winter | *S*. *arieticanis* (hay, soil),  *S*. *bertrami* (water, hay, soil),  ***S*. *bovifelis* (water, soil)**,  *S*. *capracanis* (soil),  ***S*. *cruzi* (water, 2x hay, 2x soil)**,  *S*. *miescheriana* (hay, soil),  *S*. *tenella* (water, 2x hay, soil) |
| **6** | Zarasai district | Bulls | Bulls are kept free in a large area **near the river** slope and the forest; bulls eat grass in the summer, and are fed stacked hay during the winter | *S*. *arieticanis* (2x water),  *S*. *bertrami* (water, hay),  *S*. *bovifelis* (water, hay),  *S*. *capracanis* (3x water, 2x hay, soil),  ***S*. *cruzi* (2x water, 2x hay, soil)**,  *S*. *tenella* (water, hay, soil) |
| **7** | Zarasai district | Goats | Goats are kept free in a large area, near the slope of a small lake, **without** the ability to approach it. They eat grass and stacked hay in the summer and are fed stacked hay in the winter. | *S*. *arieticanis* (water, soil),  *S*. *bertrami* (water, hay, 2x soil),  *S*. *bovifelis* (water, hay),  ***S*. *capracanis* (3x hay)**,  *S*. *cruzi* (3x water, hay),  *S*. *miescheriana* (soil),  *S*. *tenella* (water, soil) |
| **8** | Zarasai district | Sheep | Sheep are kept free in a large area **with a pond**, fed mainly with grass from the meadow. | ***S*. *arieticanis* (2x water, 2x hay, soil)**,  *S*. *bertrami* (2x water, 2x hay, soil),  *S*. *bovifelis* (2x water, hay),  *S*. *capracanis* (water, 2x hay),  *S*. *cruzi* (3x water, 3x hay, 2x soil),  *S*. *miescheriana* (soil),  ***S*. *tenella* (water)** |
| **9** | Biržai district | Cows | Cows are kept in large meadows next to cultivated fields, there is no forest nearby, the only source of water is **large puddles** of water that form in the meadows; in summer, cows are fed fresh grass, silage, hay, and in winter, concentrated feed | *S*. *arieticanis* (2x hay, soil),  *S*. *bertrami* (water, hay, soil),  ***S*. *bovifelis* (hay)**,  *S*. *capracanis* (hay),  ***S*. *cruzi* (2x water, 2x hay, soil)**,  *S*. *miescheriana* (water, hay) |
| **10** | Biržai district | Cows | Cows are kept in large meadows next to cultivated fields, there is no forest nearby, the only source of water is **large puddles** of water that form in the meadows; in summer, cows are fed fresh grass, silage, hay, and in winter, concentrated feed | *S*. *arieticanis* (water, 2x hay, soil),  *S*. *bertrami* (water),  ***S*. *bovifelis* (hay)**,  *S*. *capracanis* (2x hay),  ***S*. *cruzi* (2x water, 3x hay, soil)**,  *S*. *tenella* (hay) |

**Table S2.** The genetic identification of *Sarcocystis* species with domestic ungulates as their intermediate hosts

| Species | GenBank  accession  number | Sequence  length,  bp | Genetic similarity comparing sequences obtained in this work | |
| --- | --- | --- | --- | --- |
|  |  |  | with sequences of the same species available in GenBank | with sequences of the most closely related species |
| *S*. *arieticanis* | PV033819–PV033821 | 325 | 92.6–100%* | 87.2–87.8% *S*. *hircicanis* |
| *S*. *bertrami* | PV033822–PV033824 | 338 | 92.6–99.7% | 81.3–82.5% *S*. *asinus* |
| *S*. *bovifelis* | PV033825–PV033827 | 361 | 97.8–100% | 93.1–94.5% *S*. *bovini* |
| *S*. *capracanis* | PV033828–PV033830 | 284 | 97.2–100% | 90.8–93.6% *S*. *tenella* |
| *S*. *cruzi* | PV033831–PV033833 | 218 | 95.4–100% | 90.4–90.8% *S*. *levinei* |
| *S*. *miescheriana* | PV033834–PV033836 | 315 | 97.1–100% | 74.6–75.4% *S*. *suihominis* |
| *S*. *tenella* | PV033838–PV033840 | 296 | 96.3–100% | 90.2–93.2% *S*. *capracanis* |

* 98.5–100% comparing with *S*. *arieticanis* isolates from Europe

**Table S3.** *Sarcocystis* *hominis* *cox1* haplotypes obtained from environmental samples in Lithuania

| >H1_identified_in_eight_samples  CCACTCAGTACCAATATCGGGACTATGAATACGGAGGCCGTTGACTGGGTAATTCTTGGCCTGTTGTTCCTGGGTATGAGCAGCATCCTCGGATCCATAAACTTCCTGGGCACTAGCATGTTCGTTGGTGCTGTTGGGTCGCTGCGGTGTGGAATCCTGTATATCTGGGCAATCATATTTACCTCCATAATG |
| --- |
| >H2_ identified_in_five_samples  CCACTCAGTACCAATATCGGGACTATGAATACGGAGGCCGTTGACTGGGTAATTCTTGGCCTGTTGCTCCTGGGTATGAGCAGCATCCTCGGATCCATAAACTTCCTGGGCACTAGCATGTTCGTTGGTGCAGTTGGGTCGCTGCGGTGTGGAATCCTGTATATCTGGGCAATCATATTTACCTCCATAATG |
| >H4_idnetified_in_two_samples  CCACTCAGTACCAATATCGGGACTATGAATACGGAGGCCGTTGACTGGGTAATTCTTGGCCTGTTGTTCCTGGGTATGAGCAGCATCCTCGGATCCATAAACTTCCTGGGCACTAGCATGTTCGTTGGTGCAGTTGGGTCGCTGCGGTGTGGAATCCTGTATATCTGGGCAATCATATTTACCTCCATAATG |
| >H7_identified_in_one_sample  CCACTCAGTACCAATATCGGGACTATGAATACGGAGGCCGTTGACTGGGTAATTCTTGGCCTGTTGTTCCTGGGTATGAGCAGCATCCTCGGATCCATAAACTTCCTGGGCACTAGCATGTTCGTTGGTGCTGTTGGGTCGCTGCGGTGTGGAATCCTGTATATCTGGGCAATCATATTTACCTCCATACTG |
| >H8_identified_in_one_sample  CCACTCAGTACCAATATCGGGACTATGAATACGGAGGCCGTTGACTGGGTAATTCTTGGCCTGTTGTTCCTGGGTATGAGCAGCATCCTCGGATCCATAAACTTCCTGGGCACTACCATGTTCGTTGGTGCTGTTGGGTCGCTGCGGTGTGGAATCCTGTATATCTGGGCAATCATATTTACCTCCATAATG |
| >H9_identified_in_one_sample  CCACTCAGTACCAATATCGGGACTATGAATACGGAGGCCGTTGACTGGGTAATTCTTGGCCTGTTGTTCCTGGGTATGAGCAGCATCCTCGGATCCATAAACTTTCTGGGCACTAGCATGTTCGTTGGTGCAGTTGGGTCGCTGCGGTGTGGAATCCTGTATATCTGGGCAATCATATTTACCTCCATAATG |
| >H10_identified_in_one_sample  CCACTCAGTACCAATATCGGGACTATGAATACGGAGGCCGTTGACTGGGTAATTCTTGGCCTGTTGTTCCTGGGTATGAGCAGCATCCTCGGATCCATAAACTTTCTGGGCACTAGCATGTTCGTTGGTGCTGTTGGGTCGCTGCGGTGTGGAATCCTGTATATCTGGGCAATCATATTTACCTCCATAATG |
